# Finished Genome Assembly of *Yersinia pestis* EV76D and KIM 10v

**DOI:** 10.1128/genomeA.01024-15

**Published:** 2015-09-17

**Authors:** Shannon L. Johnson, Timothy D. Minogue, Hajnalka E. Daligault, Mark J. Wolcott, Hazuki Teshima, Susan R. Coyne, Karen W. Davenport, James G. Jaissle, Patrick S. Chain

**Affiliations:** a Los Alamos National Laboratory (LANL), Los Alamos, New Mexico, USA; b United States Army Medical Research Institute for Infectious Diseases (USAMRIID), Frederick, Maryland, USA

## Abstract

Here, we sequenced the completed genome of *Yersinia pestis* EV76D and KIM 10v, two genomes used as references in assay development, to improved high-quality draft status.

## GENOME ANNOUNCEMENT

*Yersinia pestis*, the causative agent of plague, grows as nonmotile, Gram-negative ovoid rods (0.5 to 0.8 µm by 1 to 3 µM in size) and must be grown between 23 and 28°C in order to maintain the appropriate plasmids within cells. In this project, we sequenced one isolate from each of two biovars (O and M) for whole-genome sequencing: *Y. pestis* EV76D and KIM 10v. *Y. pestis* EV76D is a clone of the EV lineage of live plague vaccines, derived from an isolate Madagascar found in the early part of the 20th century, and it is still used in some parts of the world as a vaccine strain. Conversely, *Y. pestis* KIM 10v is a Medievalis biovar variant of the KIM strain previously published (1).

Genomic DNA from *Y. pestis* EV76D and KIM 10v was extracted from purified isolates of each strain using the QIAgen Genomic-tip 500. Specifically, 100-ml bacterial cultures were grown to stationary phase and nucleic acid extracted, as per the manufacturer’s recommendations, with one minor variation. The genomic draft data include both Illumina and long-insert paired-end Roche 454 (paired insert sizes, just over 9 Kb for each) data to a total of >300× genome coverage per isolate. All raw data have been deposited in the NCBI and are available in the Sequence Read Archive (SRA) (see Table 1) (2, 3). The draft data were assembled using Newbler (version 2.6), Velvet (version 1.2.08), AllPaths (version 44837), and parallel Phrap (SPS 4.24) to generate a single closed contig of finished quality (4–7).

**TABLE 1:** Information for *Y. pestis* strains EV76D and KIM 10v

Description^a^	Data for *Y. pestis* strain:	
	EV76D	KIM 10v
WGS accession no.	LGRJ00000000	LFXP00000000
SRA accession no.	SRP06026	SRP060255
Assembly size (bp)	4,727,470	4,745,657
No. of contigs (no. of scaffolds)	20 (6)	6 (4)
No. of CDSs	4,281	4,229
% G+C content	47.6	47.7
No. of tRNAs	70	70
No. of rRNAs	18	18

a WGS, whole-genome sequencing; CDSs, coding sequences.

Each genome was assembled to just over 4.7 Mbp with >4.2 K coding sequences, 18 rRNA sequences, and 70 tRNA sequences (Table 1). Additionally, both assemblies include the three plasmids pCD, pMT, and pPCP, all of which are corroborated by orthogonal data (Wolcott, unpublished data). A preliminary review of the two annotated genomes finds several antibiotic resistance genes, consistent with independent testing (Wolcott, unpublished data), particularly to lactamase-type antimicrobials.

### Nucleotide sequence accession numbers.

The genome accession numbers to public databases are listed in Table 1.
